# Complex network analysis of thermostable mutants of *Bacillus subtilis* Lipase A

**DOI:** 10.1007/s41109-017-0039-y

**Published:** 2017-06-26

**Authors:** Nitika Kandhari, Somdatta Sinha

**Affiliations:** Centre for Protein Science Design and Engineering, Department of Biological Sciences, Indian Institute of Science Education and Research, Mohali, Punjab 140306 India

**Keywords:** Protein contact networks, Thermostability, Lipase A, Community structures

## Abstract

Three-dimensional structures of proteins that regulate their functions can be modelled using complex network based approaches for understanding the structure-function relationship. The six mutants of the protein Lipase A from *Bacillus subtilis*, harbouring 2 to 12 mutations, retain their function at higher temperatures with negligible variation in their overall three-dimensional crystallographic structures. This enhanced thermostability of the mutants questions the structure-function paradigm. In this paper, a coarse-grained complex network approach is used to elucidate the structural basis of enhanced thermostability in the mutant proteins, by uncovering small but significant local changes distributed throughout the structure, rendering stability to the mutants at higher temperatures. Community structure analysis of the six mutant protein networks uncovers the specific reorganisations among the nodes/residues that occur, in absence of overall structural variations, which induce enhanced rigidity underlying the increased thermostability. This study offers a novel and significant application of complex network analysis that proposes to be useful in the understanding and designing of thermostable proteins.

## Introduction

Proteins are biological macromolecules in the cell that are first synthesized as linear chains of amino acids held together by chemical forces, which then fold into three-dimensional structures through short and long range chemical interactions decided by the chemical nature of the amino acids (Creighton, 1992). Protein function is determined by its three-dimensional structure and significant structural change in the protein due to mutations (i.e., change in the amino acid sequence) often mediates variation in functional properties, known as the structure-function paradigm in protein science. However, there are several cases that are coming to light, where significant changes in function have been observed in some proteins due to mutations, which have negligible changes in the overall structures (Srivastava and Sinha, 2013; Srivastava and Sinha, 2014; Kandhari and Sinha, 2016; Srivastava and Sinha, 2017). Our interest has been to study those proteins and their mutants, having indistinguishable ordered crystallographic structures, but exhibiting large changes in functions. Since structure-based methods cannot easily expose the small changes, we have used the complex network approach to study these protein structures to understand the structural and mechanistic bases of what leads to the functional improvement, in absence of overall structural variations.

The three-dimensional crystallographic structure of the protein can be represented using a network description – the “Protein Contact Networks” (PCNs) - whose nodes are the amino acids (residues) and the chemical interactions between closely-held residues are the links. Such complex network based approaches have been used for studying the structure and function of proteins (Kannan and Vishveshwara, 1999; Vendruscolo et al. 2002; Bagler and Sinha, 2005; Bagler and Sinha, 2007; Barah and Sinha, 2008; Di Paola et al. 2013). In the present work, we have attempted to utilize this approach for the protein Lipase A, from the bacteria *Bacillus subtilis*, which has important applications in leather industry, food processing, pulp, and other biotechnological applications, where it needs to retain its structural integrity and functional activity at higher temperatures (at which normally proteins unfold) (Liszka et al., 2012). Six mutants of the wild-type (WT) Lipase A showing increased thermostability are chosen for this study because all the proteins have structures very similar to the WT in spite of having 2 to 12 mutations (Srivastava and Sinha, 2014; Kandhari and Sinha, 2016). The PCNs of these seven Lipase A proteins (WT and its six mutants) have been studied to uncover those small changes that do not change the structure but confer thermostability to retain functionality.

Earlier studies on thermostability have invoked the role of many structural factors that arise due to mutations and contribute to structural rigidity, such as higher number of hydrogen-bonds and salt-bridges, secondary structure stabilization, disulphide linkages, higher polar surface area, more number of Proline residues, shortening and stabilization of loops, etc. (Matsumura et al. 1989; Pjura and Matthews, 1993; Watanabe et al. 1994; Yip et al. 1995; Salminen et al. 1996; Haney et al. 1997; Russell et al. 1997; Vogt and Argos, 1997; Vogt et al. 1997; Nicholson et al. 1998; Watanabe and Suzuki, 1998; Chan et al. 2011; Gromiha et al. 2013). We have shown, using the complex network approach that thermostability in Lipase A, imbibed through several structural factors that arise due to mutations in its mesophilic counter-part, can be elaborated through the study of small changes in the contact patterns in the PCNs. We show the detailed analysis of the small conformational changes, occurring through-out the proteins, by analyzing the contact patterns, their positional information, the local network parameters, and the changes in the community memberships, with respect to their structural stability and functionality at higher temperatures.

The paper is organised as follows: Methods and Data section gives the construction of the PCNs, and the details of the protein Lipase A and its mutants. A short description of the network parameters and method of community structure analysis is mentioned here. The network analyses results are shown along with community structure analysis in the Results section. Finally, the results are discussed in relation to the protein’s structure-function correlation. This analysis demonstrates the advantage of network-based analysis in understanding small changes in protein structures that are otherwise difficult to identify, and paves the way for application of complex networks in understanding the correspondence between the structure and function of proteins that can help in design and engineering of proteins with desired functions.

## Methods and data

### Construction of protein contact networks

PCNs were constructed by considering the C-α atom of each residue in the protein as a node and the spatial proximity between any two of them as the link.

**Fig. 1:**
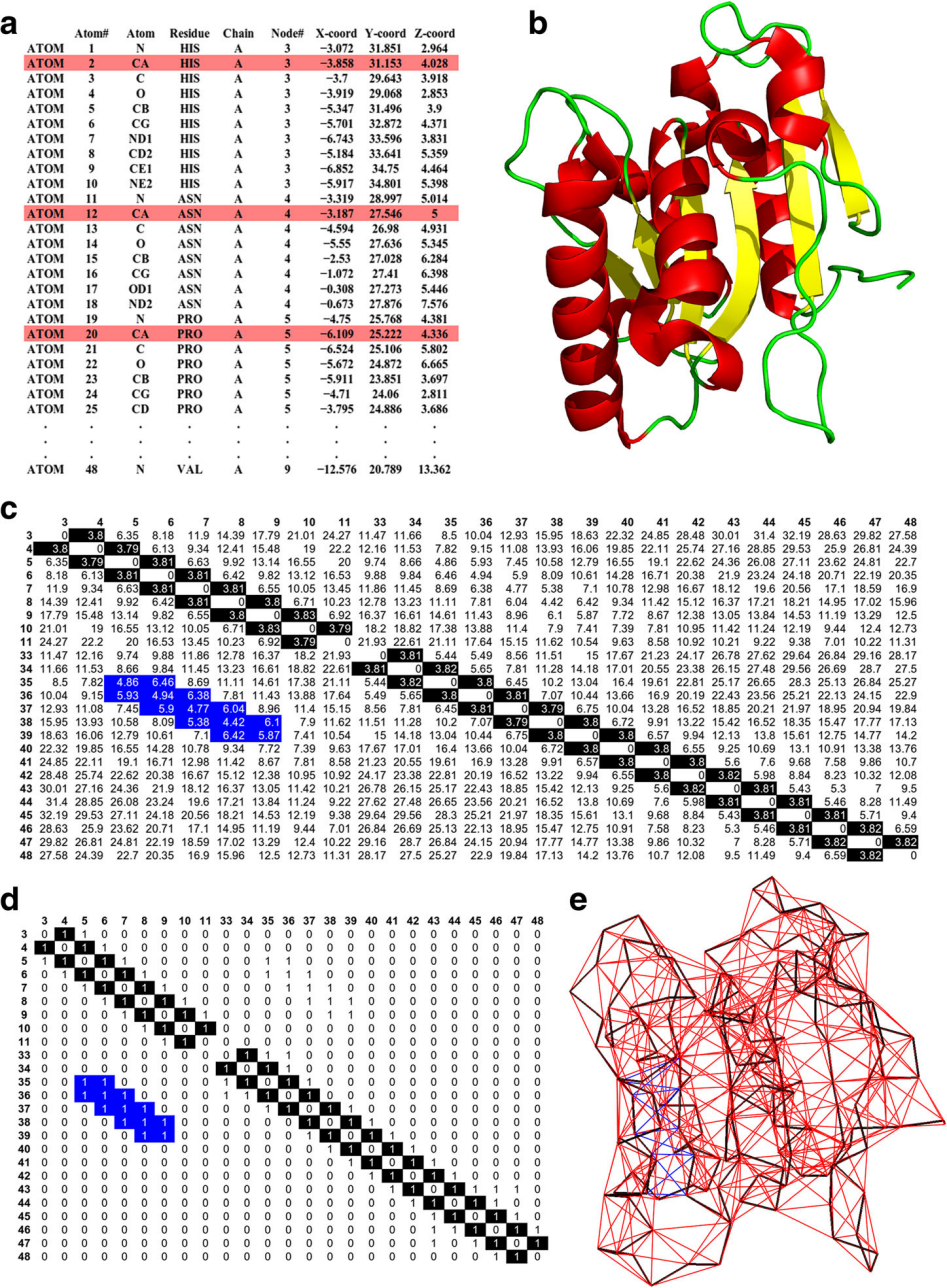
Construction of the Protein Contact Network of Lipase A. (**a**) Crystallographic coordinates of all atoms of the first 3 amino acids in the protein chain. (**b**) Three dimensional structure of WT Lipase A corresponding to the coordinates; (**c**) Pair-wise Distance matrix between portion of the C-αatoms; (**d**) Corresponding Adjacency matrix; (**e**) PCN of WT Lipase A shown in (**b**). See text for details

The Protein Data Bank (PDB) is a database for storing solved crystallographic structures of proteins (RCSB Protein Data Bank (PDB) 1971). Figure 1 gives the method of construction of the coarse-grained PCN for all seven protein structures from the PDB data containing the positions (x, y and z coordinates) of all the atoms of all amino acids in 3-dimensional space. Fig. 1a shows the crystallographic coordinates of all atoms of the first three residues of the protein WT Lipase A (shown in Fig. 1b). We use a coarse-grained approach (Bagler and Sinha 2005; Bagler and Sinha 2007; Srivastava and Sinha 2014) to construct the network by considering the spatial positions of only the protein backbone atoms (i.e. C-α atoms) of each residue (coloured in red in Fig. 1a; the backbone connections are highlighted in black in Fig. 1c and d). A pair-wise Euclidean distance matrix is computed between the C- α atoms of all residues (Fig. 1c). The distance matrix is converted into an Adjacency matrix, A (Fig. 1d), using the following rule –

$$ {\mathrm{A}}_{\mathrm{ij}}{=1,\mathrm{if}\ \mathrm{D}}_{\mathrm{ij}}{<=7 \AA,\mathrm{else},\mathrm{A}}_{\mathrm{ij}}=0 $$

where, D_ij_ is the Euclidian distance between C-α atoms of the i^th^ and j^th^ residues. This cut-off has been widely used for constructing C-α based Protein Contact Networks, and provides a reasonable estimate of the interacting residue pairs in the first interaction shell (Haliloglu et al. 1997). However there are other cut-offs that have been used in all atom network models (Gromiha and Selvaraj 2004). It is obvious that there will be changes in the number of edges if the cut-off is varied. For C-α based PCNs, the most commonly used values are 0.7 nm to 0.8 nm (Bagler and Sinha 2005; Bagler and Sinha 2007; Srivastava and Sinha 2014; Srivastava and Sinha 2017). The results presented in this work have been checked with 0.75 nm and 0.80 nm cut-offs, and they are comparable to the results presented here.

The two-dimensional representation of the coarse-grained PCN, corresponding to the three dimensional structure of Lipase A (Fig. 1b), is shown in Fig. 1e. The black lines in Fig. 1e are the back-bone contacts, the red lines are the contacts between the other nodes present throughout the protein, the and the blue lines (in Fig. 1c and d) are long range contacts between pairs of nodes that are far apart in the linear chain but have come closer in space due to protein folding (e.g., contacts between nodes 5–36, 6–36, 9–39).

### The data-set: Lipase A and its mutants from *Bacillus subtilis*

**Fig. 2:**
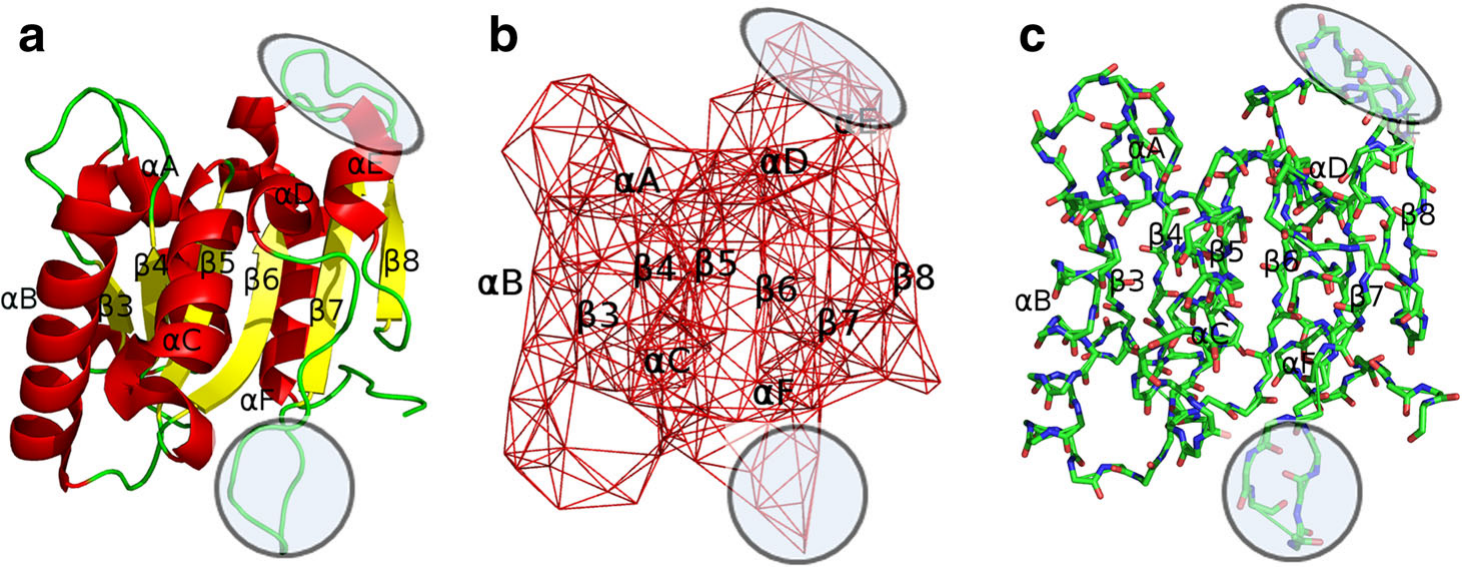
Schematic representation to show the correspondence between protein secondary structures to the PCN. The α-helices are denoted by α, and the β-sheets by β

The high resolution X-ray crystallographic structures of WT Lipase A (shown in Fig. 2a) and its mutants from *Bacillus subtilis*, obtained from the Protein Data Bank, shows a compact minimal α/β hydrolase fold with a six-stranded parallel β-sheet (β3 to β8 in yellow) flanked by six helices (α-A to α-F in red), two α-helices on one side and three α-helices and a 3_10_ helix on the other (Van Pouderoyan et al. 2001). It has a globular shape with a length of 179 amino acids, and carries out the hydrolysis of fats and esters. The functional sites can be divided into two parts: the catalytic triad and the active site. The catalytic triad, as the name suggests, is made up of 3 residues, Ser 77, Asp 133 and His 156, which brings about the main lipase activity of the protein. The active site consists of 14 residues (Ile 12, Ala 15, Phe 17, Asn 18, Met 78, Ala 105, Leu 108, Met 134, Ile 135, Leu 140, Gly 155, Ile 157, Leu 160 and Tyr 161), which support the lipase activity carried out by the catalytic triad. In Fig. 2, are shown the WT Lipase A structure in two representations: Ribbon representation (Fig. 2a), and Ball and Stick model (Fig. 2c). The PCN corresponding to this protein is shown in Fig. 2b. The correspondence between the different secondary structures (α-helices, β-sheets, loop regions, etc.) in Fig. 2a and c are circled to compare with the contacts between the nodes in the PCN (Fig. 2b).

Our dataset from PDB comprising of Lipase A wild-type (WT) protein and its six thermostable mutants are shown in Table 1. These mutants, with 2–12 mutations, were obtained in a manner so that the previous mutations are preserved and new mutations are incorporated (Acharya and Rao 2004; Ahmad et al. 2008; Kamal et al. 2011). All the mutant proteins are stable and functional at increasingly higher temperatures.

**Table 1:** The PDB structure IDs of Lipase A and its six mutants used for analysis, with the mutated sites and their optimum functional temperature

PDB ID	Mutations	Optimum Temperature( °C)
1I6W (WT)	WILD-TYPE	35
1T4M (M1)	**N166Y, A132D**	--
1T2N (M2)	N166Y, A132D, **L114P**	45
3D2A (M3)	N166Y, A132D, L114P, **I157M**	--
3D2B (M4)	N166Y, A132D, L114P, I157M, **F17S, N89Y**	50
3D2C (M5)	N166Y, A132D, L114P, I157M, F17S, N89Y, **A15S, A20E, G111D**	55
3QMM (M6)	N166Y, A132D, L114P, I157M, F17S, N89Y, A15S, A20E, G111D, **M134E, M137P, S163P**	65

New mutations are highlighted in BOLD

### Network parameters

**Degree** of a node is the total number of direct links that it has to other nodes. For PCN all backbone nodes, except the terminal nodes, have a minimum degree of 2 since they connect the protein chain. Larger degree indicates secondary structures and long range contacts due to folding of the chain.

**Shortest Path Length (SPL)** between a pair of nodes is the smallest number of links that need to be traversed in order to reach from one node to the other. It has been shown that fibrous and globular protein and helices and sheets have different path lengths (Bagler and Sinha 2005).

**Clustering Coefficient (CC)** measures the cliquishness of the neighbourhood of the node, i.e. to what extent the nodes in a network tend to cluster together. It is defined as the ratio of the number of edges among the linked neighbours of the node to the total number of edges possible amongst them. It has been shown that globular proteins and nodes in helices have higher CC compared to fibrous proteins and sheets (Bagler and Sinha 2005).

**Betweenness Centrality (BC)** of a node is the number of shortest paths passing through that node. More the number of paths passing through a node, more crucial it is for communication between different parts of the network.

**Closeness Centrality (CCen)** is defined as the inverse of the sum of Shortest Paths from that node to all other nodes in the network. High CCen of a node indicates closeness to other nodes.

For more details see (Newman 2010).

## Community structure analysis

Finding communities or modules in a network attempts to divide the network nodes into groups, such that there is a higher density of edges within the groups than between them. Community structure analysis was performed for all the seven PCNs using the fast greedy algorithm (Clauset et al. 2004).

Protein data extraction from PDB was done using in-house PERL scripts. Network analysis and visualization have been done with CYTOSCAPE (Shanon et al. 2003). All statistical analysis was done using R (https://cran.r-project.org/).

## Results

**Fig. 3:**
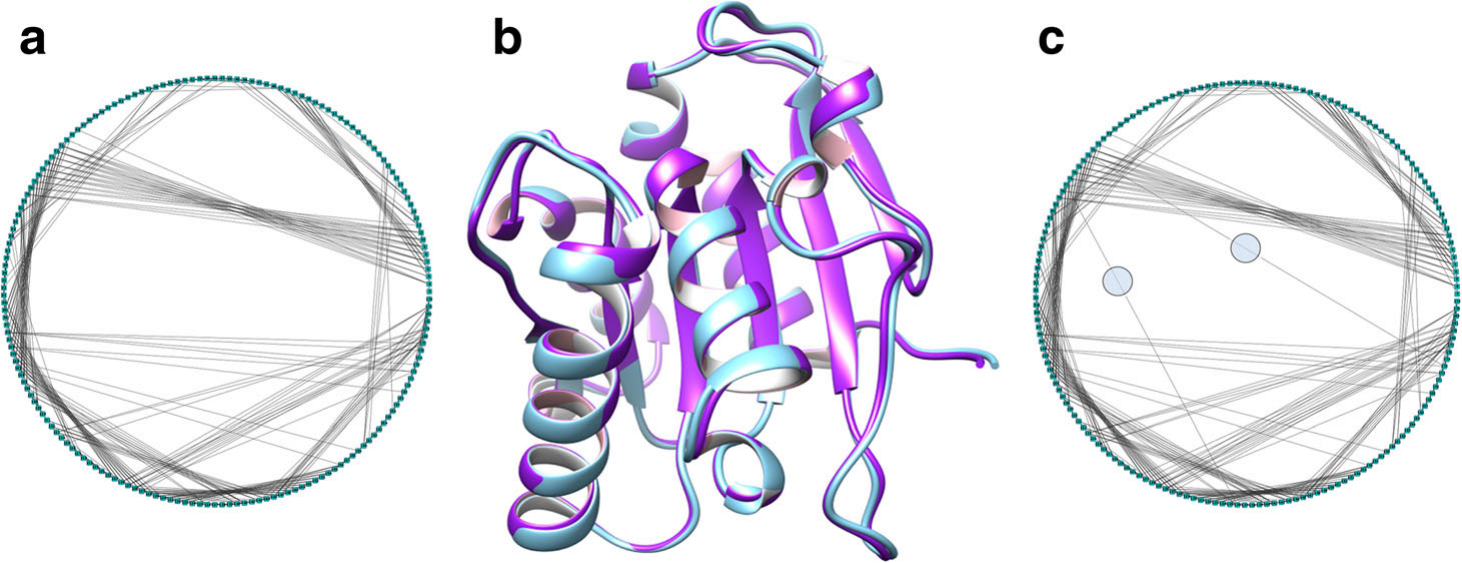
Network and structural representations of WT and M6: Ring Graph representation of the PCNs of (**a**) WT and (**c**) M6. (**b**) Three-dimensional structural overlap of WT (*blue*) and M6 (*purple*) proteins

The three-dimensional structures of the seven Lipase A proteins have been found to be very similar in spite of having 2–12 mutations (Srivastava and Sinha 2014; Kandhari and Sinha 2016). The cross structure Root Mean Square Deviation (RMSD), which is an indicator of average difference between pairs of structures, is negligible and ranges between 0.18 Å–0.39 Å for all seven structures (Srivastava and Sinha 2014). In absence of significant structural variations, we analysed the coarse-grained PCNs to reveal small differences in contacts among the nodes, as shown in Fig. 3. Figure 3b shows the two overlapping crystallographic structures of WT (blue) and the last mutant M6 (purple), with cross-structure RMSD only 0.39 Å – suggesting very high structural overlap despite 12 mutations and high thermostabilty. Figures 3a and c shows the ring graph representations of WT and M6, where the nodes in the linear protein chain are positioned as a ring and the short and long-range contacts among them are shown in black lines. The ring graphs highlight the long range contacts among the nodes, which come closer on folding of the linear chain in the three-dimensional structure. Comparison of Fig. 3a and c easily reveal small changes in contacts (circles in Fig. 3c). In the following section, the results are organised to highlight these local variations in the PCNs of the seven protein structures using complex network analysis, and elucidate the changes in the structures that may have been responsible for their enhanced thermostability.

### Mutant protein networks have larger number of contacts

**Fig. 4:**
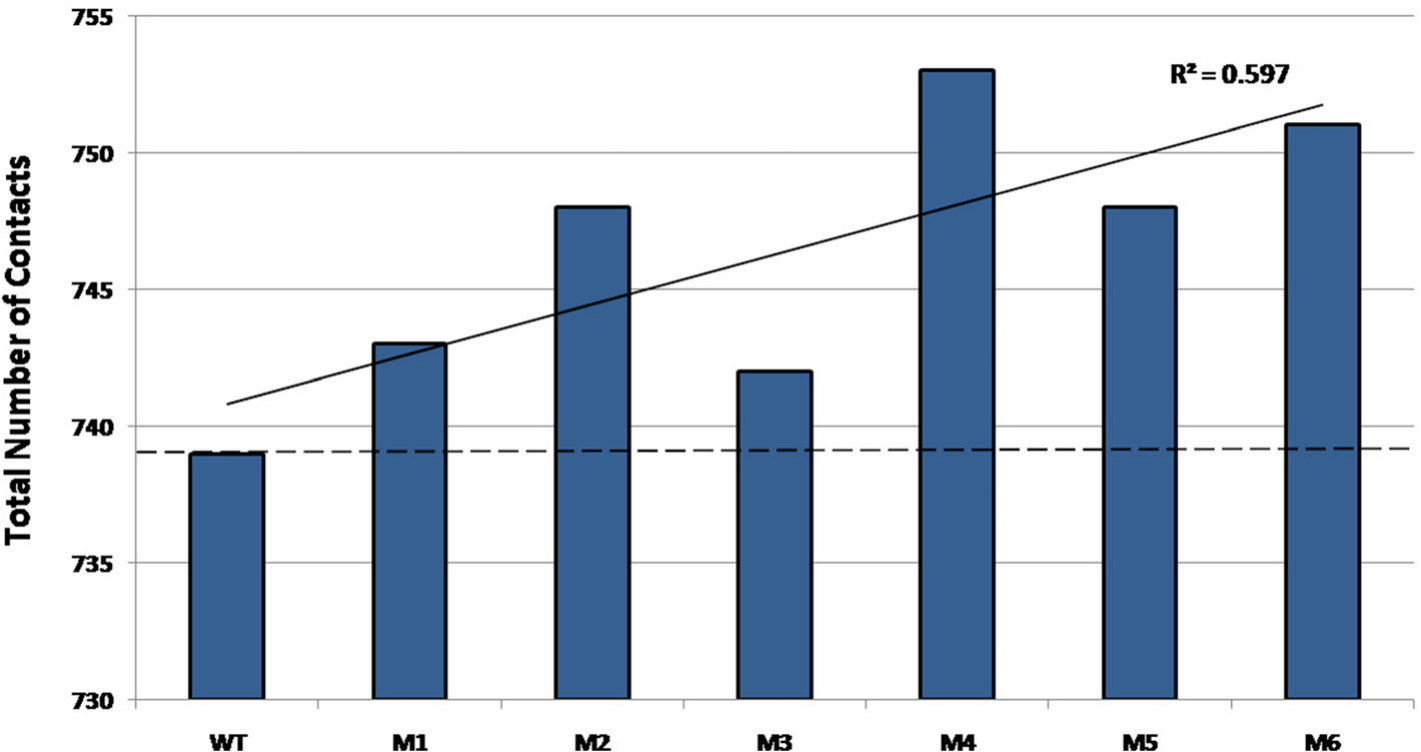
Total number of contacts (edges) in the PCNs of the WT and mutant Lipase A. The *trend line* shows an increasing tendency with coefficient of determination R^2^ ~ 60%

No change in the structures at global scale despite the change in function points towards careful analysis at the local scale. Even though the total number of nodes in all PCNs is the same, the number of contacts (edges) in each PCN, shown in Fig. 4, indicates variation. New contacts are formed and old ones lost due to mutations. All the mutant PCNs have larger number of contacts compared to the WT, and the trend line shows that there is a general increase in the number of edges in mutants with increased thermostability (black solid line). Even though this increase does not directly correlate with increased thermostability (e.g., M4 has the largest number of edges (753) but is not the most thermostable among the mutants), this result indicates that larger number of contacts may introduce higher rigidity in the mutant proteins. This raises the question as to how these contacts are distributed in the PCNs, which can lead to thermostability.

### Network parameter analysis of PCNs corroborate negligible structural variations

Increase in the total number of edges in the mutant PCNs can influence the network parameters. The five network parameters studied for the seven PCNs are - Degree, Betweenness Centrality (BC), Shortest Path Length (SPL), Clustering Coefficient (CC) and Closeness Centrality (CCen). These were first studied at a global-scale i.e. the average network parameters were computed for each PCN. The ranges for average network parameter values for the seven PCNs are:

$$ \mathrm{Degree}:\kern0.5em 8.291{\textstyle \hbox{-} }8.413 $$

$$ \mathrm{Betweenness}\ \mathrm{Centrality}:0.0180\hbox{-} 0.0184 $$

$$ \mathrm{Closeness}\ \mathrm{Centrality}:0.240\hbox{-} 0.244 $$

$$ \mathrm{Clustering}\ \mathrm{Coefficient}:0.527\hbox{-} 0.539 $$

$$ \mathrm{Shortest}\ \mathrm{Path}\ \mathrm{Length}:4.173\hbox{-} 4.239 $$

The very low variations of the average network parameters in all PCNs reflect the low cross-structural RMSD of the proteins, thereby corroborating similar three-dimensional structures of Lipase A and its thermostable mutants.

**Fig. 5:**
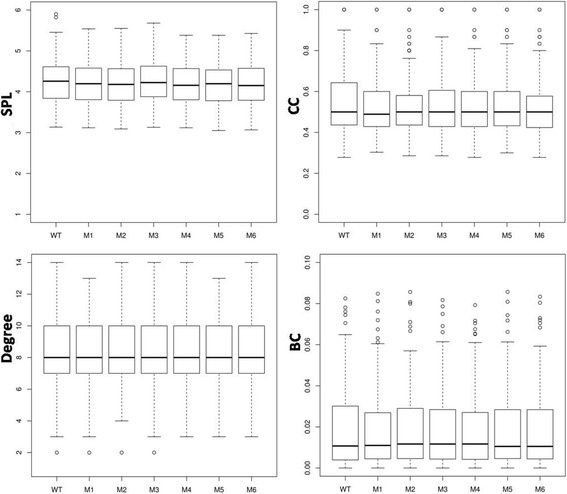
The *box plots* of network parameters (SPL, CC, Degree and BC) for the seven PCNs (WT and M1-M6)

To find any local changes, the network parameters (SPL, CC, Degree, and BC) were computed at individual node level in all seven PCNs (WT, and M1 to M6), and the details of their distributions are shown in the box-plots in Fig. 5. For all parameters, the distributional characteristics of all network parameters seem to be similar for all PCNs in that group (i.e., WT and its mutants) with small variations in the extreme values. If the distributions are skewed, then all PCNs show the same behaviour (see Degree and BC). For Degree, the distributional variation is the least among all PCNs. The box-plot for CC shows that, even though the median values are similar, all mutant PCNs have smaller box size and more outliers compared to the WT. This indicates that most nodes in the mutant PCNs have CC values close to their average/median value, but they also have more nodes with higher CC. The Kruskal-Wallis test for the null hypothesis of equal means show no significant difference (*p* < 0.01) in the means of all PCNs for all network parameters. These results point towards careful analysis of local contacts in each mutant that may be responsible for increased thermostability.

### Contacts analysis reveals their specific roles in imparting stability to the mutant proteins

The known factors attributed to increasing stability/rigidity in proteins that are important for thermostability are - loop stabilization (Vogt and Argos 1997), stabilization of secondary structures (Nicholson et al. 1998), stabilization of termini contacts, higher number of hydrogen bonds (Vogt and Argos 1997; Vogt et al. 1997; Pjura and Matthews 1993), shortening of loops (Vogt and Argos 1997; Nicholson et al. 1998), higher polar surface area, higher number of disulphide linkages (Matsumura et al. 1989) and salt bridges (Yip et al. 1995; Chan et al. 2011; Russell et al. 1997; Nicholson et al. 1998), increased buried surface area after oligomerization (Salminen et al. 1996), increase in number of proline residues (Watanabe et al. 1994; Watanabe and Suzuki 1998), surrounding hydrophobicity (Gromiha et al. 2013), etc. A detailed analysis of the new contacts made in the six PCNs of the mutant proteins reveals their contribution into stabilizing some of these factors (listed in Table 2). It is clear that these contacts are mostly stabilizing the loops and the active site region in the mutants.

**Table 2:** New contacts in PCNs contribute to the stabilizing factors in mutant proteins

	M1	M2	M3	M4	M5	M6
Loop stabilization	√√	√√√	√√	√√	√√	√√√
Secondary Structure Stabilization	√√	√√	√√	√√	√√	√√
Termini stabilization	√	√	√	√	√	√
Active site Rigidity	√√	√√	√√	√√	√√	√√

Ticks correspond to the number of contacts involved in each factor: √ for 0–6 contacts; √√ for 6–12 contacts; and √√√ for 12–18 contacts

### Community structure analysis of WT PCN

The overall structural similarity of the mutant proteins and few changes in contacts in their PCNs (Fig. 4) that contribute to specific stabilizing factors aiding to thermostability (Table 2) prompted a detailed study in the changes in the community structures in the seven PCNs. It has been shown that modules in PCNs aid in efficient information transfer between different protein domains (Del Sol et al. 2007). Changes in contacts among the nodes can result in reorganisation of the communities thereby influencing information transfer. This comparative analysis allows a better understanding of the small but distributed changes that helps in retaining functionality at higher temperatures (thermostability) in the mutant proteins.

**Fig. 6:**
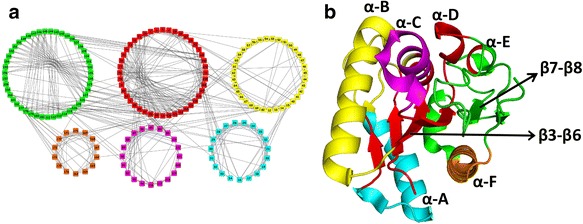
Community structure analysis of the wild-type (WT) PCN. (**a**) Six major communities in WT; (**b**) Three-dimensional structure of the WT Lipase A. *Colour code* in B corresponds to the communities in A

The WT Lipase A protein, shown in Fig. 6b with the known secondary structures (helices: α-A to α-F and β-strands: β3 to β8), consists of six communities (Fig. 6a). It is clear that the communities correspond to the major secondary structures of the protein and clearly shows the structural regions that are closely connected. The communities in the WT are: **Red** (having the central β-sheet: β3, β4, β5, β6; helix α-D and part of helix α-E); **Green** (the largest module with portions of helices α-E and α-F, strands β7 and β8, and major loop region); **Yellow** (the helix α-B, a small portion of helices α-C and terminal α-A); **Cyan** (a major portion of helix α-A); **Magenta** (lower portion of the helix α-C), and the smallest community **Orange** (portion of α-F). In the rest of the community structure analysis for the mutant PCNs, these colours of the member nodes have been kept the same for easy identification of their reorganization. The loop regions where many functional sites, including the catalytic triad, exist and mutations were made in the thermostable mutants, are primarily in the Green community in WT PCN.

### Community structure analysis unravels increased stability in the mutants

**Fig. 7:**
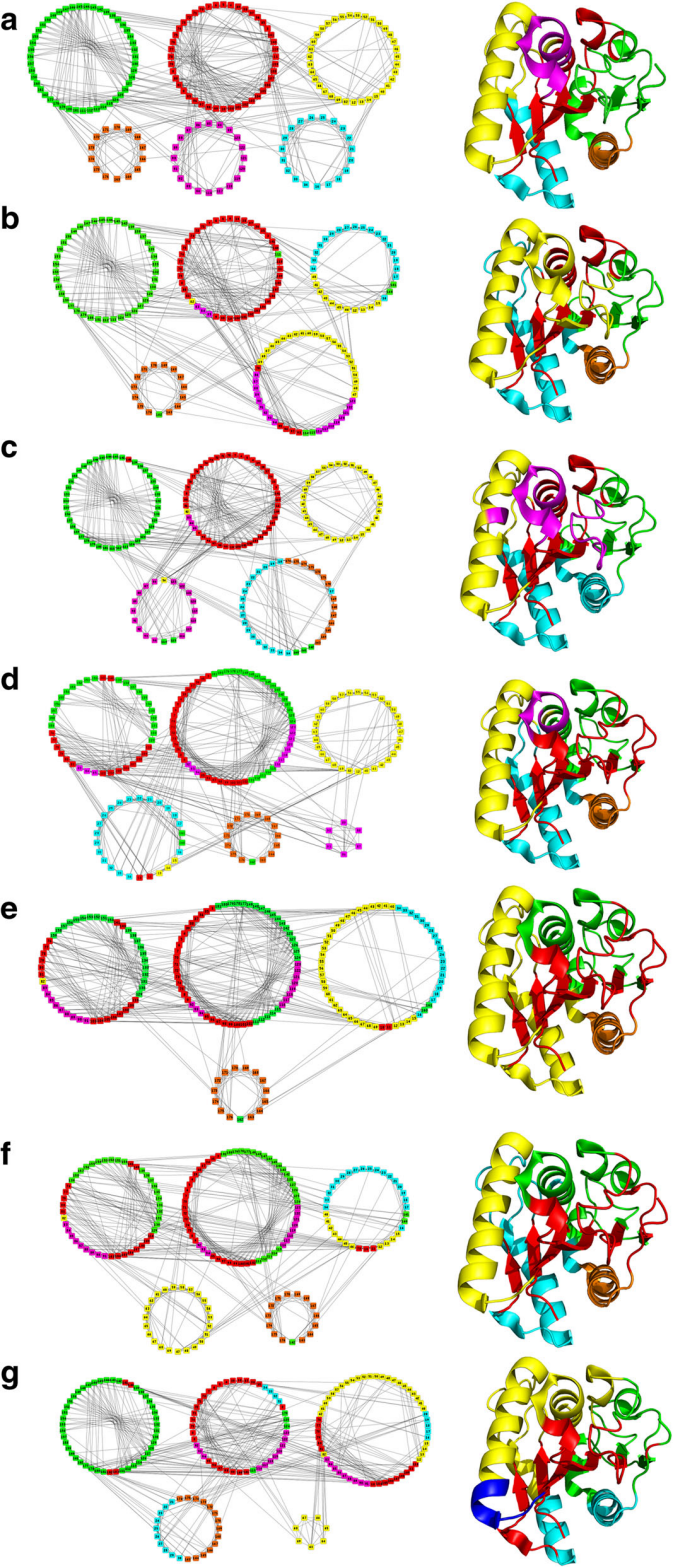
Reorganisation of communities in WT and six mutant PCNs and their structures. *Colour-coding* is as per the WT modules. See text for details

Change in contact patterns in different mutant PCNs can result in changes in their community structures. Figure 7 shows the community structures for all seven Lipase A PCNs. The six mutant PCN communities (Fig. 7(b-g)) reveal changes with respect to the WT PCN communities (Fig. 7a) that arise due to the changed contact patterns occurring due to 2 to 12 mutations in the mutant proteins (M1 to M6). The corresponding crystallographic structures for the proteins are also shown along with their communities with same colour code. It is clear from Fig. 7 that the few changes in contacts among the mutant PCNs result in quite a few differences in community memberships among the PCNs. The major secondary structures (α-helices and β-sheets) generally remain in single communities keeping the integrity and retaining the similarity of the protein structures, but the nodes that fluctuate among communities in different PCNs can be identified and correlated with the contacts made/broken - to lead to the thermostability in the mutants. The loop regions are relatively flexible in the protein structure and are expected to change modules. In some cases, the terminal nodes of the regular secondary structures also changed their community if the adjoining loop had changed its community.

The number of communities in WT PCN is the largest (6). The PCN of M3 also shows 6 communities but one of them is very small (only 6 nodes). Rest of the mutants have 4 or 5 communities. This is indicative of the networks becoming more compact, as within the same space the nodes are having larger groupings. For proteins this signifies that the larger groups of residues being more connected among them-selves confer more rigidity to the structures, even when the motions of the atoms increase at higher temperatures.

Below we analyse the communities in the mutant PCNs, vis-à-vis structural regions in the proteins, in Fig. 7 that gives some useful information.

#### Red module

This module contains nodes primarily involving the central β-sheet, which forms the core of Lipase A structure. Figure 7(b-g) shows that the four β-strands β3, β4, β5 and β6) of the central β-sheet forming a single community in WT (Fig. 7a) maintain its integrity in all the mutants. In addition, the Red module is seen to widen as more nodes from adjacent β-strands and the lower loop region come together into one community leading to *loop stabilization* in mutants. Thus, the community that forms the protein core “expands” thereby strengthening the core of the protein and thus imparting stability to the protein’s functional region in all thermostable mutants.

#### Yellow module

The nodes in this community stay constant in most mutant PCNs maintaining the integrity of the secondary structure – the α-B helix. For M2, only one node merges with another module without breaking into smaller modules indicating presence of new contacts. In M6, the two contacts (48–54 and 72–173) tend to pull the helix α-B in opposite directions, thus making the terminal part (nodes 63 to 67) of the helix form a different community altogether (coloured Blue with nodes 63–69 in the structure of M6 in Fig. 7g). In this mutant (M6), the nodes containing the helices α-B, α-C and α-D form a single community (Yellow) leading to *secondary structure stabilization*. Recently several point mutations (N51F, G52 M, V54H, L55F, F58I, V59I in helix α-B) belonging to our Yellow module and I87W in helix α-C were predicted that leads to increase in contacts between helices imparting stability to them and thus causing delay in thermal unfolding (Rathi et al. 2015; Rathi et al. 2016). These results were shown and validated experimentally for few of these mutations (Rathi, et al. 2016). So, stabilization of helices in the Lipase A structure, as shown in our community structure analysis, is crucial for protein stability at higher temperatures.

#### Cyan module

The nodes in this community also stay constant in most mutant PCNs maintaining the integrity of the secondary structure (helix α-A), except in M4 and M6 where it becomes members of the Yellow community (fully or partially) indicating *secondary structure stabilization.*

#### Green module

The nodes in the loop regions, that are part of the Green module in WT, change communities and become part of the Red community that stabilizes the central β-sheet structure in M3-M4-M5-M6, or joins the Yellow community through *loop stabilization* and imparts stability to M1. The small helix α-E, which otherwise was split into Red and Green communities, in WT and first two mutants (M1 and M2), integrates completely into the green module along with the loop regions and terminal α-F region in M3 and other more thermostable mutant PCNs. This leads to *loop stabilization* and reduced number of modules of the PCN inducing compactness.

#### Orange module

The orange module consists of nodes 163 to 174. The nodes corresponding to helix α-F (156 to 174) retains its structural integrity in all PCNs but shows *secondary structure stabilization* with the Cyan module α-A in some mutants. The other few nodes, being part of loops, change community memberships.

#### Magenta module

In WT (Fig. 7a), this module includes nodes containing a portion of helix α-C and nearby loop regions. The nodes of this community change their membership to the other modules in the mutant PCNs extensively often leading to *loop stabilization*, even though the integrity of the helix is maintained in all PCNs with the nodes staying together always. The contact 85–90 is important for the formation of this module. Whenever this contact appears, the Magenta module appears. In case of M3, the formation of contacts 48–81 and 52–83 pulls nodes 81, 82 and 83 away from the 86 to 91 stretch. Also, the contacts 88–92 and 91–94 lead to formation of a tiny separate module comprising of only 6 nodes (86 to 91).

#### Catalytic triad residues

Among these three important functional residues, Asp 133 and His 156 remains in the Green community throughout, but Ser 77 changes its community from Red (in WT, M1, M2) to Green (in M3, M4, M5) to Yellow (in M6). These changes in community membership of Ser 77 (in the loop region) is associated with *active site rigidity* and more effective functioning at higher temperature, such as, binding of 77 and 156 during hydrolysis, which has been shown experimentally (Kamal et al. 2012).

In addition to the above, our analysis of the modules show that, owing to the other mutations in M6, there are more number of contacts surrounding the α-helices α-D and α-E and the central β-sheet (giving rise to a single module consisting of yellow, cyan, magenta and red nodes in Fig. 7g), which further stabilize M6. This is also supported by results from the structural experiments (Rathi et al. 2015; Kamal et al. 2012).

#### Information transfer between different modules in the protein

In WT, of the 17 functional nodes taking part in Lipase A activity, 11 nodes connect the Red, Green Yellow and Cyan communities among themselves. On the other hand, in M6 mutant PCN only 7 out of the 17 functional residues are involved in connecting different modules. This clearly suggests that there is less information transfer through the functional residues among the different parts of the protein. This is useful when less transfer of perturbation is needed within the protein for stability at higher temperature.

The results from the comparative analysis of these community structures of WT and six mutant PCNs clearly identifies and correlates the stabilizing role of the few but distributed contacts made/broken through loop stabilization, secondary structure stabilization, and active site rigidity, thereby making the mutants more thermostable.

## Discussion

In this study we use the complex network approach to study the protein Lipase A and its six mutants, having 2 to 12 mutations (all at or near the functional sites), from *Bacillus subtilis*, which do not have any significant difference in their three-dimensional structure, but are functional at very high temperatures (i.e. thermostable). There are two important issues that have been addressed in this paper. First, we attempt to understand the small changes arising in these proteins due to mutations, which are not easily identifiable using standard structural analysis due to their having similar structures. Secondly, we endeavour to identify those localised changes distributed throughout the proteins that bring about more stability/rigidity in the mutant proteins to be functional at higher temperatures.

The coarse-grained PCN approach is suitable to study this problem as it highlights any change at the node-level contacts in these thermostable mutants that may alter information transfer within the protein structures. Since the over-all crystallographic structures of all seven proteins are similar, the global network parameters are not useful indicators of variations in their thermostabilty. Through a careful and detailed comparative analysis of the contacts in each of the PCNs, along with their community structure analyses, we have identified what may be the underlying structural correlates for increased thermostability in the mutants of Lipase A. On a careful enumeration of the contacts made and broken in the six mutants PCNs, the total number of contacts show a small but consistent increase in the mutants in comparison to the WT Lipase A. Through a detailed position-specific analyses of these new contacts we could clearly associate them to stabilize the loop regions, secondary structures (forming inter-helical contacts), and in making the active site region more rigid. These features, as mentioned in the introduction, have been attributed to increase structural stability in proteins.

The community structure analysis of the modules in the mutant PCNs succinctly reveals the significant changes in the residue memberships that correlate with overall structural rigidity. Our results also showed reduction in the number of modules, and reorganization of modules through merging of nodes among the three major modules, Red, Green and Yellow, in the mutants. The Red module, involving the central beta sheets that give stability to the protein, merges with the Green module in which the functional catalytic triad residues are present in the loop region, along with the Yellow module which possesses the helix α-B that is intact in all mutants. Reduction in the number of communities and the reorganization of their nodes - both can help in reducing inter-modular spread of perturbation at higher temperature keeping the structure of the proteins similar, and also allow enhanced intra-modular communication among residues involving larger parts of the proteins for increased rigidity at higher temperature. Both contribute to make the proteins more compact in the mutants, thus, imparting stability and increased activity at higher temperature (Rathi et al. 2015; Kamal et al. 2012). This kind of comparative community structure analysis to unravel structural features of protein is both novel and interesting with far-reaching consequences in understanding the role of small and distributed allosteric changes to protein function.

Taking cue from our results, one can study the dynamic consequences, using molecular dynamics simulations (Karplus and McCammon, 2002), of the group of residues that constitute the communities and specifically those which change modules often, to speculate their role in imparting thermostability in Lipase A. Thus, this complex network based approach can be very useful in design and engineering of specific properties in proteins.

## Abbreviations

ASPL: Average Shortest Path Length
BC: Betweenness Centrality
CC: Clustering Coefficient
CCen: Closeness Centrality
M1: First Mutant (1T4M)
M2: Second Mutant (1T2N)
M3: Third Mutant (3D2A)
M4: Fourth Mutant (3D2B)
M5: Fifth Mutant (3D2C)
M6: Sixth Mutant (3QMM)
PCN: Protein Contact Networks
PDB: Protein Data Bank
RMSD: Root Mean Square Deviation
SPL: Shortest Path Length
WT: Wild-type protein (1I6W)
